# Community-acquired cutaneous ulcer in a child caused by *Serratia marcescens*

**DOI:** 10.1099/jmmcr.0.003228

**Published:** 2014-12-01

**Authors:** Vineeta Sharma, Archana Angrup, Santwana Verma, DigVijay Singh, Anil Kanga

**Affiliations:** Department of Microbiology, Indira Gandhi Medical College, Shimla, Himachal Pradesh, India

**Keywords:** *Serratia marcescens*, cutaneous ulcer, ceftazidime

## Abstract

**Introduction:**

*Serratia marcescens* is a Gram-negative, aerobic, motile bacillus belonging to the family *Enterobacteriaceae*. *S. marcescens* has been implicated in different types of infections including urinary tract infection, septicaemia, meningitis and wound infections. Very few cases of skin infections caused by this organism have been reported in the medical literature. *S. marcescens* is an important nosocomial pathogen but has rarely been implicated as a cause of community-acquired soft-tissue infections.

**Case presentation:**

We present a rare case of a community-acquired spontaneous cutaneous ulcer in an immunocompetent child from a sub-Himalayan region. Infections caused by *S. marcescens* may be difficult to treat because of its ability to produce a β-lactamase, which confers resistance to broad-spectrum, β-lactam antibiotics.

**Conclusion:**

In our patient, the treatment was modified to ceftazidime and amikacin after sensitivity testing and the patient’s condition improved. This necessitated isolation by culture and antimicrobial susceptibility testing to ensure appropriate therapy.

## Introduction

*Serratia marcescens* is a Gram-negative, aerobic, motile bacillus belonging to the family *Enterobacteriaceae*. It is found in water and soil, on plants and in animals. The first description of infection caused by *S. marcescens* was given in 1951 at Stanford hospital (Wheat *et al.*, 1951). *S. marcescens* has been implicated in different types of infections including urinary tract infection, septicaemia, meningitis and wound infections. Very few cases of skin infections caused by this organism have been reported in the medical literature. The infections are predominantly observed in immunocompromised patients or in those with pre-damaged skin (Langrock *et al.*, 2008). *S. marcescens* is an important nosocomial pathogen but has rarely been implicated as a cause of community-acquired soft-tissue infections. The portal of entry of community-acquired *S. marcescens* skin infections has not been clearly described in most previous reports (Friedmann *et al..*, 2003). Here, we present a rare case of a community-acquired spontaneous cutaneous ulcer in an immunocompetent child from a sub-Himalayan region.

## Case report

**Fig. 1.**
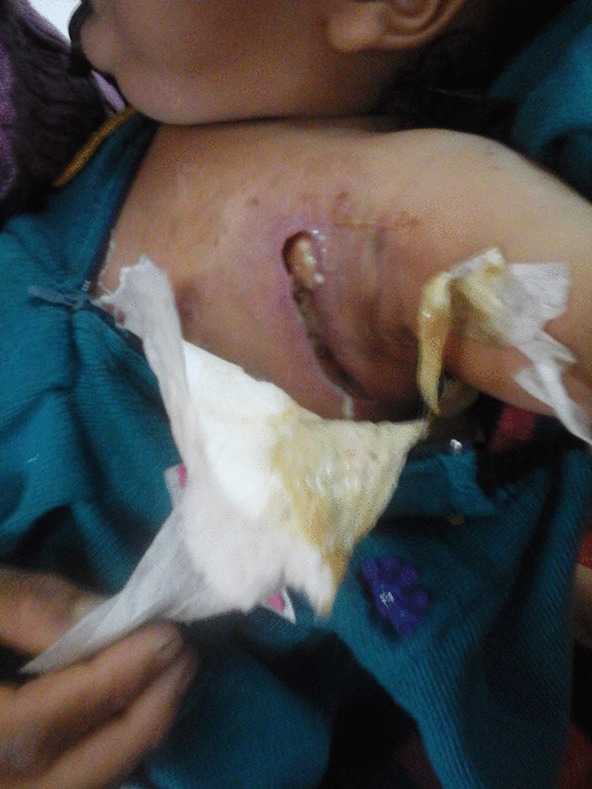
Ulcer measuring about 8 cm in length by 3 cm in width and 2–3 cm in depth. The base contained pus and necrotic tissue.

A female child, aged 11 months, was admitted to the Department of Paediatrics with a history of fever and an ulcerated lesion on the upper left side of the chest. According to her mother, the child was apparently well 5 days previously when she noticed a redness over the left side of chest on the upper part. The mother gave a history of insect bite at that site followed by a history of itching. There was an area of redness that gradually increased in size with formation of pustules that burst on the third day discharging a thick yellow pus. On local examination, there was an ulcer measuring about 8 cm in length by 3 cm in width and 2–3 cm in depth. The base contained pus and necrotic tissue. The base was soft and was not attached to the underlying structures. The margins of the cavity were regular and non-indurated (Fig. 1).

On physical examination, the pulse rate was 102 min^−1^, respiratory rate was 28 breaths min^−1^ and body temperature was 100 °F. There was no lymphadenopathy. Other systemic examinations were within normal limits. Laboratory tests showed the following results: total leucocyte count 9960 µl^−1^ with 40 % neutrophils, 52 % lymphocytes, 5 % monocytes and 3 % eosinophils; haemoglobin 9.6 g dl^−1^; platelets 454000 µl^−1^; urea 10 mg dl^−1^; creatinine 0.5 mg dl^−1^; sodium 131 mmol l^−1^; potassium 4.7 mmol l^−1^; total protein 6.1 g dl^−1^; and erythrocyte sedimentation rate 60 mm in the first hour. C-reactive protein was also raised with a value of 83.08 l^−1^. The immunization history of the child was complete up to 9 months of age and a BCG scar was present. Developmental milestones were normal. The mother was non-reactive for human immunodeficiency virus types 1 and 2.

A clinical diagnosis of a left-sided chest wall ulcer subsequent to a burst abscess was made. Chest X-ray was done, which showed a normal parenchyma and bony structures. The patient was initially started on injected ampicillin plus cloxacillin 200 mg intravenously (IV) every 6 h along with metronidazole 50 mg IV every 8 h and amikacin 120 mg IV once a day, keeping in mind possibility of a polymicrobial infection. The patient did not show much improvement as a result of the empirical treatment.

Pus from the lesion was inoculated onto blood and MacConkey agar and incubated at 37 °C. Growth of a lactose-non-fermenting organism on MacConkey agar and grey colonies on blood agar after 24 h incubation was obtained. On Gram staining of the colonies, Gram-negative bacilli were seen, which were motile in the hanging drop preparation. Biochemical reactions were negative for indole, methyl red, urease and citrate utilization tests. Triple sugar iron agar showed an alkaline/acid reaction without gas formation.

**Fig. 2.**
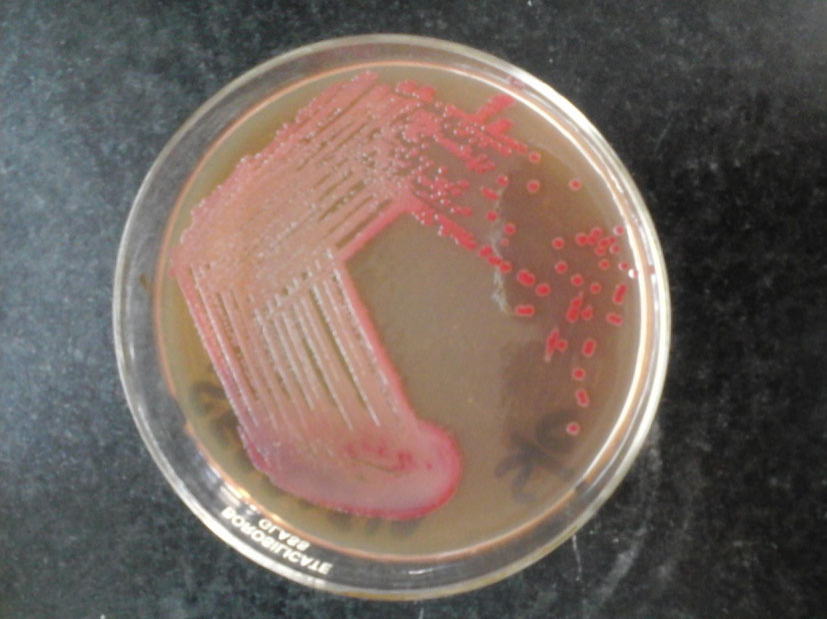
MacConkey culture plate showing the production of a red-coloured pigment characteristic of *Serratia.*

**Fig. 3.**
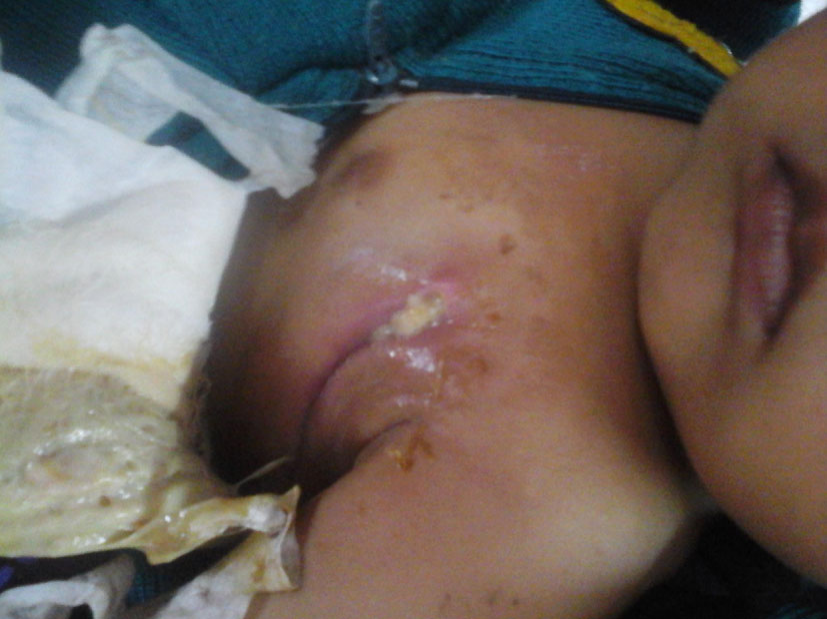
Lesion showing signs of healing in the form of healthy granulation tissue, a decrease in size and approximation of the margins.

The following day, the primary culture plate day showed the production of a red-coloured pigment, which is characteristic of *Serratia* (Fig. 2). For further speciation, l-arabinose and sucrose fermentation tests were carried out and were positive. An ornithine decarboxylase test was positive, with negative results for an arginine decarboxylase test. The organism was finally identified as *S. marcescens*. The organism was sensitive to ceftazidime, amikacin, imipenem and amoxyclav, and was resistant to ampicillin, piperacillin/tazobactam and netilmicin. A Mueller–Hinton agar sensitivity plate also showed the production of a red-coloured pigment (Fig. S1, available in the online Supplementary Material). A blood culture done to rule out septicaemia was sterile. After the sensitivity report, metronidazole and ampicillin plus cloxacillin were omitted and intravenous ceftazidime (400 mg every 8 h at a rate of 150 mg kg^−1^ day^−1^) and amikacin (120 mg IV once a day) was given for 10 days. Under this therapy the lesion started to show signs of healing in the form of healthy granulation tissue, a decrease in size and approximation of the margins, as shown in Fig. 3. There was no pus discharge and no slough. The patient was discharged on amoxyclav 5 ml twice a day. On follow-up after 2 weeks, the lesion healed completely.

## Discussion

*S. marcescens* was originally considered to be a non-pathogenic saprophytic water organism. Infections caused by *Serratia* have been reported with increased frequency since 1960. This prodigiosin-producing bacterium is found in various ecological niches. It can survive in hostile conditions such as nutrient-poor reservoirs and disinfectant, and thus acts as a nosocomial agent (Soria *et al.*, 2008). However, there are only a few case reports of community-acquired infections caused by this organism.

The various infections reported to be caused by *S. marcescens* are pneumonia, bacteraemia, urinary tract infection, endocarditis, meningitis and infections of the musculoskeletal system. Skin and soft-tissue infections are rarely reported. The clinical manifestations of skin infections include granulomatous lesions, nodules, dermal abscesses, ulcers, cellulitis and necrotizing fasciitis. (Munoz–Perez *et al.*, 1996) even reported a case of disseminated papular eruption in human immunodeficiency virus-positive patients. Skin infections occur mostly in immunocompromised patients or those with co-morbidities. Of the 10 patients with necrotizing fasciitis, several co-morbidities and risk factors were noted such as haemodialysis for advanced renal disease, corticosteroid use, diabetes and chemotherapy (Rehman *et al.*, 2012). In a case report by (Langrock *et al.,* 2008), the patient had multiple abscesses on the right leg with chronic venous insufficiency and long-term immunosuppresion due to low-dose corticosteroid therapy.

Most of the soft-tissue infections by *S. marcescens* have no definite portal of entry, although various authors have suggested that previous injury, animal bites and ulcers can act as routes of entry (Bogaert *et al.*, 1991; Grim *et al.*, 2010). In our case, we presume that the child had itching after an insect bite, which damaged the skin and acted as the portal of entry. The child was immunocompetent and there was no predisposing factor or any associated co-morbidities. A similar finding was observed by (Giráldez *et al.,* 2011), who reported a case of purulent abscesses on the dorsum of the hand by *S. marcescens* in a 40-year-old immunocompetent man.

Infections caused by *S. marcescens* may be difficult to treat because of its ability to produce a β-lactamase, which confers resistance to broad-spectrum, β-lactam antibiotics, often complicating the therapy. Aminoglycosides show good activity against this organism, but resistant strains have also been reported (Hejazi & Falkiner, 1997). In our patient, the treatment was modified to ceftazidime and amikacin after the sensitivity testing and the patient’s condition improved. This necessitated isolation on culture and antimicrobial susceptibility testing to ensure the appropriate therapy.

## Abbreviations

IV: intravenous
